# CRISPR-Cas9-mediated elimination of plasmid-borne carbapenemase genes restores ertapenem susceptibility in clinical *Klebsiella pneumoniae* isolates

**DOI:** 10.1016/j.bj.2026.100966

**Published:** 2026-03-16

**Authors:** Shu-Fang Kuo, Tsung-Yu Huang, Chih-Yi Lee, Fang-Ju Chen, Chen-Hsiang Lee

**Affiliations:** a Department of Laboratory Medicine, Chiayi Chang Gung Memorial Hospital, Chiayi, Taiwan; b Department of Medical Biotechnology and Laboratory Sciences, College of Medicine, Chang Gung University, Taoyuan, Taiwan; c Division of Infectious Diseases, Department of Internal Medicine, Chiayi Chang Gung Memorial Hospital, Chiayi, Taiwan; d School of Medicine, College of Medicine, Chang Gung University, Taoyuan, Taiwan; e Microbiology Research and Treatment Center, Chiayi Chang Gung Memorial Hospital, Chiayi, Taiwan; f Division of Infectious Diseases, Department of Internal Medicine, Kaohsiung Chang Gung Memorial Hospital, Kaohsiung, Taiwan

**Keywords:** Genome editing, Antimicrobial resistance, Insertion sequence

## Abstract

**Background:**

Carbapenem-resistant *Klebsiella pneumoniae* (CRKP) represents a critical public health threat due to its broad-spectrum antimicrobial resistance and capacity for horizontal gene transfer.

**Methods:**

Three clinical CRKP isolates, each carrying one of the three major classes of carbapenemase as class A (*bla*_KPC_), class B (*bla*_NDM_), and class D (*bla*_OXA_) were selected. A CRISPR/Cas9-based system (pCasKP-pSGKP) was employed to target carbapenem resistance genes in these strains (KP21040 with *bla*_OXA-181_, KP4-78 with *bla*_NDM-1_, and KP5-4 with *bla*_KPC-2_).

**Results:**

CRISPR/Cas9-mediated editing led to partial reduction or complete loss of resistance plasmids, as evidenced by S1 nuclease-pulsed-field gel electrophoresis. This plasmid elimination correlated with a marked restoration of susceptibility to ertapenem, showing a greater than 64-fold reduction in minimum inhibitory concentrations (MICs) across all strains. In KP21040, MICs for ertapenem and levofloxacin decreased to 0.006 μg/mL and 0.125 μg/mL, respectively. Whole-genome analysis revealed that *bla*_OXA-181_ was flanked by insertion sequence (IS)*26* elements, which mediated homologous recombination upon CRISPR-induced double-strand breaks, resulting in excision of a ∼15 kb segment including *bla*_OXA-181_ and *qnrS1*. These findings suggest that ISs may enhance CRISPR efficacy by promoting recombination-driven deletion. Moreover, the complete removal of all three resistance plasmids was observed in the KP5-4 strain harboring *bla*_KPC-2_.

**Conclusion:**

This study demonstrates that CRISPR/Cas9-based genome editing can eliminate plasmid-encoded carbapenemase genes in clinical CRKP isolates and, in specific genetic contexts, facilitate the concurrent removal of associated quinolone resistance determinants. These findings support CRISPR-based genome editing as a proof-of-concept strategy for addressing plasmid-mediated multidrug resistance in Gram-negative pathogens.

## Highlights

• A clustered regularly interspaced palindromic repeats was used to modify carbapenem resistance genes in clinical carbapenem-resistant *Klebsiella pneumoniae* isolates. It effectively restored carbapenem susceptibility and may provide a framework to assess plasmid-associated gene functions in clinical isolates.

## 1 Introduction

*Klebsiella pneumoniae* is a major cause of severe infections, particularly in immunocompromised patients [1]. The emergence of carbapenem-resistant *K. pneumoniae* (CRKP) poses a serious clinical challenge due to resistance to carbapenems, which are often last-line antibiotics for severe infections. This resistance is largely mediated by plasmid-encoded carbapenemase genes that hydrolyze β-lactam antibiotics and are frequently located on mobile genetic elements, facilitating horizontal transfer and co-dissemination with other resistance determinants [2,3]. In the face of limited therapeutic options and a declining antibiotic pipeline, innovative approaches such as plasmid curing have gained attention as potential strategies to eliminate carbapenemase genes while preserving bacterial community integrity [4].

Clustered regularly interspaced palindromic repeats (CRISPR) and associated Cas proteins form an adaptive immune system in bacteria that provides sequence-specific defense against plasmids and bacteriophages [5]. The CRISPR-Cas9 system employs guide RNA to direct Cas9-mediated double-strand DNA cleavage, a property that has been widely adapted for programmable genome editing [6,7]. In bacteria, engineered CRISPR-Cas9 systems can selectively eliminate resistance genes or plasmids, restoring antibiotic susceptibility and potentially limiting the spread of antimicrobial resistance determinants [8,9].

In this study, we used the CRISPR (pCasKP-pSGKP) system for target DNA recognition and Cas9 protein generation, resulting in blunt-ended double-strand breaks to remove plasmids with resistance genes in clinical CRKP isolates [10,11]. Moreover, insertion sequences (ISs) facilitate horizontal gene transfer across diverse species. IS transposition into the CRISPR-Cas system occurs during non-homologous end-joining repair, while genome mining reveals IS disruption of other prokaryotic defense mechanisms. Previous studies have highlighted the crucial role of ISs in undermining CRISPR-Cas and other genetic defenses in prokaryotes [12,13]. We conduct comprehensive sequencing of resistance plasmids to elucidate their genetic structure, including ISs, and to clarify resistance plasmid propagation among clinical CRKP strains after CRISPR-Cas intervention.

## 2 Materials and methods

### 2.1 Experimental strains, DNA extraction and PCR detection of carbapenemase genes

We selected three clinical CRKP isolates, each representing one of the major Ambler classes of carbapenemase such as class A (*bla*_KPC_), class B (*bla*_NDM_), and class D (*bla*_OXA_) [3,14] to encompass clinically relevant resistance mechanisms. Strains KP21040, KP4-78, and KP5-4 were obtained from the microbiology laboratory of Chang Gung Memorial Hospital. Species identification was confirmed using the MALDI-TOF MS Biotyper system (Bruker Microflex LT/SH, Bruker Daltonics GmbH). Antimicrobial susceptibility testing was performed with the BD Phoenix M50 system (BD Diagnostic Systems, Sparks, MD) using the GN-NMIC411 panel, and results were interpreted according to Clinical and Laboratory Standards Institute (CLSI) M100 guidelines [15]. CR was defined as resistance to at least one carbapenem, with breakpoints of MIC >2 μg/mL for ertapenem, MIC >4 μg/mL for imipenem, or MIC >4 μg/mL for meropenem. These three CRKP strains DNA extraction were conducted using Qiagen DNeasy Tissue Kits. The primers used to target various carbapenemase genes are detailed in [Table 1]. Sequence comparisons were performed using the BLAST tool available on the NCBI website (http://www.ncbi.nlm.nih.gov).

**Table 1:** The primers for carbapenem resistance gene and CRISPR.

Target gene	Primer name	Sequence	Function	Reference
*bla*_OXA-48_	OXA-48-like-F	TGTTGCTGAAGGAGTTGGGC	Amplification of the *bla*_OXA-48_ from *Klebsiella pneumoniae* (K21040) strain genome	[14]
	OXA-48-like-R	AGCAGCGCATAGTCATTTGC		
*bla*_KPC_	blaKPC-F	TGTTGCTGAAGGAGTTGGGC	Amplification of the *bla*_KPC_ from *Klebsiella pneumoniae* (KP5-4) strain genome	[14]
	blaKPC-R	ACGACGGCATAGTCATTTGC		
*bla*_NDM_	blaNDM-1-F	TAAAATACCTTGAGCGGGC	Amplification of the *bla*_NDM_ from *Klebsiella pneumoniae* (KP4-78) strain genome	[14]
	blaNDM-1-R	AAATGGAAACTGGCGACC		
-	M13-R	CAGGAAACAGCTATGACC	pSGKP M13 primer	[11]
*Cas9*	Cas9-SQ4	GTTAAAATCCGTTAAAGAGTTACTAGG	Amplification of the Cas9 from pCasKP plasmid	This study
	CAT-R	GCAACTGACTGAAATGCCTC		

### 2.2 Multilocus sequence typing (MLST)

The three clinical CRKP strains were analyzed using MLST [16,17] with seven specific genes. The reaction primers are detailed in [Supplementary Table 1. The sequences of PCR products were compared with the Institute Pasteur database to assign seven allelic numbers, which facilitated the determination of the MLST sequence type.

### 2.3 Plasmids, primers, and construction of CRISPR plasmids

The plasmids used in the CRISPR experiments are shown in [Supplementary Table 2], and the primers are shown in [Table 1]. For antibiotic selection, 30 μg/mL apramycin (apr+) and 50 μg/mL kanamycin (km+) were used. Two plasmid systems, pCasKP-apr and pSGKP-km, were employed for CRISPR/Cas9-mediated gene editing. The plasmids pCasKP-apr (Addgene plasmid #117231, RRID:Addgene_117231) and pSGKP-km (Addgene plasmid #117233, RRID:Addgene_117233) were obtained from Addgene (http://n2t.net/addgene:117231 and http://n2t.net/addgene:117233, respectively). The pCasKP-apr plasmid contained the *rpsL* promoter for *Cas9* expression, an l-arabinose-inducible *P*_*araB*_ promoter for lambda red genes, and a temperature-sensitive replicon (repA101ts). The pSGKP-km plasmid contained sgRNA, J23119 promoter, and *sacB* gene for plasmid elimination. Repair of the broken DNA was facilitated by homologous recombination directed by lambda red.

### 2.4 Editing of carbapenemase genes

The CRISPR system was used to modify the *bla*_OXA-181_, *bla*_NDM-1_, and *bla*_KPC-2_ genes [11,18]. The pSGKP plasmid was linearized with the *Bsa*I enzyme in a specified reaction mixture. Following a 3-h incubation at 37 °C, gel electrophoresis confirmed linearization of the plasmid. For designing oligonucleotides targeting specific genes, protospacer adjacent motif (PAM) sites were identified. A 20 bp spacer sequence was synthesized adjacent to each PAM site and three distinct spacer sets were created, as detailed in [Table 2] and [Supplementary Figs. 1–4]. To ligate oligos into the sgRNA expression plasmid (pSGKP-km), the reaction mixture comprised linearized sgRNA plasmid, diluted annealed oligos, T4 DNA Ligase buffer, T4 DNA Ligase, and ddH_2_O. The ligation process was facilitated by overnight incubation at 16 °C in a PCR machine. The vector (pSGKP) was thawed on ice, and *E. coli* DH5α was retrieved from −80 °C storage. After thawing, 0.5 μl of the vector was placed into a microcentrifuge tube with *E. coli* DH5α. Subsequently, the mixture underwent heat shock at 42 °C for 45 s, and the tube was then placed back on ice for 2 min. The heat-shocked bacterial suspension was then added to pre-warmed LB broth and incubated at 37 °C with shaking for 1 h. Following incubation, 100 μl of transformed cells were plated onto LB agar containing kanamycin and incubated overnight at 37 °C.

**Table 2:** The design for sgRNA and editing efficiency.

Target gene	sgRNA name	Sequence	Function	Editing efficiency (%) (n = 40)	Reference
*bla*_OXA-181_	sgRNA OXA-F1	TAGTCACCAAGTCTTTAAGTGGGA	Forward, OXA-181 spacer for gene deletion	97.5%	[18]
	sgRNA OXA-R1	AAAC TCCCACTTAAAGACTTGGTG			
*bla*_KPC-2_	sgRNA KPC-F1	TAGTCCAGCTCCCAGCGGTCCAGA	Forward, KPC-2 spacer for gene deletion	95.0%	[11]
	sgRNA KPC-R1	AAACTCTGGACCGCTGGGAGCTGG			
*bla*_NDM-1_	sgRNA NDM-F1	TAGTCCCAACGGTGATATTGTCAC	Forward, NDM-1 spacer for gene deletion	95.0%	[18]
	sgRNA NDM-R1	AAACGTGACAATATCACCGTTGGG			

Electroporation was performed at 2.5 kV with a resistance of 200 Ω and a capacitance of 25 μF, resulting in a time constant of approximately 5 ms. For the first electroporation, the pCasKP-apr plasmid was introduced into the selected strains (KP21040, KP4-78, and KP5-4). The bacterial cultures were then centrifuged at 4 °C at 8000×*g* for 10 min to eliminate the supernatant. The pellet was washed with cold sterile water followed by 10% ice-cold glycerol, and then centrifuged again at 4 °C at 8000×*g* for 10 min. Finally, the pellet was placed into a 50 mL centrifuge tube and washed with 10% ice-cold glycerol. For the second electroporation, 50 μl of competent cells (KP21040, KP4-78, and KP5-4 strains, which already carry pCasKP-apr) and 5 μl of pSGKP-km were combined in a pre-cooled Eppendorf tube. The mixture was then transferred to a cuvette for electroporation at specified settings. Following electroporation, the cells were incubated in LB broth without antibiotics for 1.5 h. The cells were centrifuged, and the supernatant was removed, leaving a residual solution. The pSGKP-km plasmid was introduced into strains already containing pCasKP-apr. The residual cell suspension was plated onto LB agar containing apramycin and kanamycin and incubated overnight. After incubation, 40 colonies were randomly selected and subjected to PCR analysis to confirm loss of the targeted carbapenem resistance genes or plasmids. Editing efficiency was calculated as the percentage of correctly edited clones among the total number of colonies screened. After successful gene editing, plasmid curing was conducted to eliminate both plasmids from the strains. The strains were cultured on LB plates with 5% sucrose and incubated at 37 °C. Selected colonies were then inoculated onto LB plates with and without antibiotics to verify plasmid removal. Strains in which both plasmids had been successfully eliminated were expected to grow only on plates devoid of antibiotics.

### 2.5 Whole plasmid next-generation sequencing

Selected strains underwent next-generation sequencing [19]. Plasmids containing carbapenemase resistance genes were extracted using Qiagen Plasmid Maxi Kits. Sequencing of the plasmids was performed using Illumina's NovaSeq X Plus system. Gene assembly was conducted using Spades v3.15.5., and plasmid replicon types were identified through PlasmidFinder (https://cge.food.dtu.dk/services/PlasmidFinder/). Plasmid sequences were uploaded to Proksee (https://proksee.ca/), an expert system for bacterial genome assembly, annotation, and visualization. Using its built-in annotation engine, gene features were identified, and both circular and linear plasmid genome maps were generated with interactive graphical outputs.

### 2.6 S1 nuclease-PFGE analysis

The analysis of plasmid size and quantity was conducted utilizing pulsed-field gel electrophoresis (PFGE) on the Bio-Rad CHEF Mapper system subsequent to treatment with 50 Units of S1 nuclease (Thermo Scientific™, Lithuania). The parameters for electrophoresis were established as follows: switch time ranging from 7 to 35 s, an angle of 120°, a voltage gradient of 6 V/cm, maintained at a temperature of 14 °C for a duration of 15 h. The Lambda Ladder PFGE Marker served as the molecular size reference standard. The gel underwent staining with 15 μL of ethidium bromide (10 mg/mL) for a duration of 30 min, and the resultant DNA band patterns were visualized and digitally recorded employing the AlphaEase™ imaging system (Alpha Innotech Corporation, San Leandro, CA). The S1-PFGE technique was employed to evaluate the plasmid curing effects on designated strains.

### 2.7 Antibiotic susceptibility testing

Susceptibility to ertapenem and levofloxacin was tested using the E-test (bioMérieux) according to the manufacturer's instructions. Determination of the minimum inhibitory concentration (MIC) was based on CLSI clinical breakpoints M100 34th Edition [15].

## 3 Results

### 3.1 Carbapenemase genes and MLST analysis

PCR confirmed the presence of *bla*_OXA-181_ in KP21040, *bla*_NDM-1_ in KP4-78, and *bla*_KPC-2_ in KP5-4. MLST identified KP21040 as ST15, KP4-78 as ST307, and KP5-4 as ST16. These strains represent clinically prevalent CRKP lineages, commonly associated with plasmid-borne carbapenemase genes, and reflect distinct genetic backgrounds and phylogenetic diversity.

### *3.2* *CRISPR/Cas editing of bla*_OXA-181_*, bla*_NDM-1_*, and bla*_KPC-2_*genes*

Following the introduction of the pCasKP and pSGKP plasmids, we successfully eliminated plasmids carrying *bla*_OXA-181_, *bla*_NDM-1_, and *bla*_KPC-2_, as confirmed by PCR. The editing efficiency was greater than 95% (shown in [Table 2]).

### 3.3 Plasmid changes and antibiotic susceptibility test results after CRISPR/Cas9 intervention

Depending on plasmid architecture and genetic context, CRISPR/Cas9 targeting resulted in either recombination-mediated partial deletion of resistance gene–containing regions or complete loss of resistance plasmids, as confirmed by S1 nuclease–pulsed-field gel electrophoresis [Fig. 1]. KP21040 contained two plasmids, estimated at 160 kb and 50 kb. After CRISPR/Cas9 treatment, two target-cured strains (A1 and A17) were identified. Compared to the original KP21040 strain, the ∼50 kb plasmid in the target-cured strains was diminished to approximately 35 kb. In addition, *bla*_OXA-181_ was absent in these target-cured strains, indicating its localization on the ∼50 kb plasmid. The CRISPR/Cas9 system did not eliminate completely the plasmid harboring *bla*_OXA-181_, but rather removed an approximately 15 kb fragment of the gene, which included *bla*_OXA-181_ [Fig. 1]. For the KP4-78 strain, S1-PFGE analysis revealed the presence of three plasmids. The absence of one plasmid was found in the target-cured strains (B1 and B2) post CRISPR treatment, and *bla*_NDM-1_ was not detected, indicating its location on the removed plasmid, approximately 55 kb in size. In the KP5-4 strain, the target-cured strains (C1 and C2) retained only one plasmid after CRISPR treatment, reflecting the elimination of three larger plasmids, approximately 80 kb, 110 kb, and 200 kb in size, and *bla*_KPC-2_ was also not detected. Confirmation via whole plasmid sequencing was required to ascertain whether all three eliminated plasmids harbored *bla*_KPC-2_, prompting their detection and removal by the CRISPR system.

**Fig. 1:**
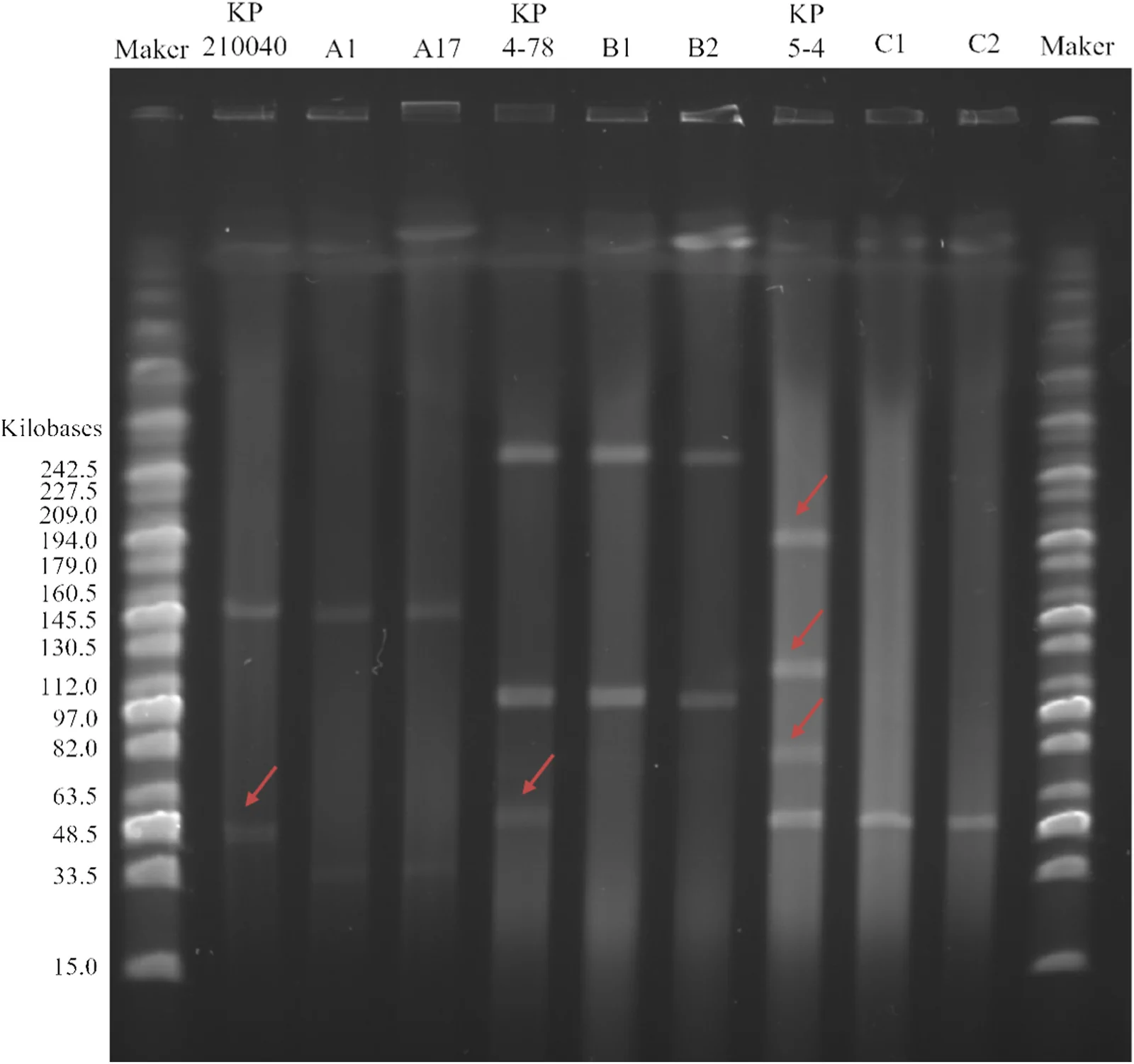
S1-PFGE analysis of plasmid profiles before and after CRISPR/Cas9 treatment. The original strain KP21040 harbored a plasmid of approximately 50 kb (indicated by the red arrow). After CRISPR editing, the plasmid bands in the target-cured strains A1 and A17 were markedly reduced in size, suggesting partial plasmid deletion. These results indicate that the *bla*_OXA-181_ gene was located on the ∼50 kb plasmid and was only partially excised. A1 and A17 are independent target-cured derivatives obtained from two separate CRISPR editing experiments on the same parental KP21040 strain, and sequencing/PCR mapping confirmed that the deleted regions were identical in both derivatives. Similarly, KP4-78 carried a ∼55 kb plasmid (red arrow), which was absent in the target-cured strains B1 and B2 following CRISPR intervention. PCR analysis confirmed the loss of the *bla*_NDM-1_ gene in these strains. KP5-4 originally contained three plasmids of approximately 80 kb, 110 kb, and 200 kb (red arrows). All three plasmids were eliminated in the target-cured strains C1 and C2, and *bla*_KPC-2_ was no longer detectable by PCR.

The antibiotic susceptibility test results are depicted in [Supplementary Fig. 5]. Antimicrobial susceptibility assessments revealed that the original strain KP21040 displayed a resistant phenotype characterized by a MIC of 2 μg/mL for ertapenem and 1.5 μg/mL for levofloxacin. Conversely, its genetically modified target-cured strain (A1) exhibited expanded inhibition zones and a significant reduction in MICs, thereby reinstating susceptibility to both ertapenem and levofloxacin. Strains KP4-78 and KP5-4 demonstrated resistance to ertapenem, with MICs ≥32 μg/mL. Following the application of CRISPR/Cas9 technology, the target-cured strains B1 and C1 both exhibited markedly diminished MICs (0.5 μg/mL), indicating a restoration of susceptibility to ertapenem.

### 3.4 Analysis of genetic structural changes after CRISPR/Cas9 editing

Whole plasmid sequencing of strain KP21040 identified a 50,542 bp plasmid, designated p2140OXA-181, classified as IncX3 [Supplementary Fig. 6]. This plasmid harbored two resistance genes—*bla*_OXA-181_ and *qnrS1*—flanked by insertion sequences (IS*26*, truncated IS*3000*, and IS*Kpn19*). Comparative analysis with NCBI databases revealed 100% sequence identity to several plasmids disseminated among Enterobacteriaceae, indicating its widespread occurrence. After CRISPR/Cas9 intervention, target-cured strains A1 and A17 retained p2140OXA-181 but exhibited a reduction in plasmid size from ∼50 kb to ∼35 kb, were identical in both derivatives [Fig. 1]. PCR mapping and sequencing revealed a deleted segment of 14,124 bp containing *bla*_OXA-181_ and *qnrS1* [Fig. 2]. The deleted region was flanked by IS*26* on both sides, suggesting that Cas9-induced double-strand breaks triggered IS*26*-mediated homologous recombination, excising the intervening segment. This recombination event likely allowed the plasmid to evade complete elimination while removing the central resistance gene cluster, thereby restoring susceptibility to ertapenem and levofloxacin.

**Fig. 2:**
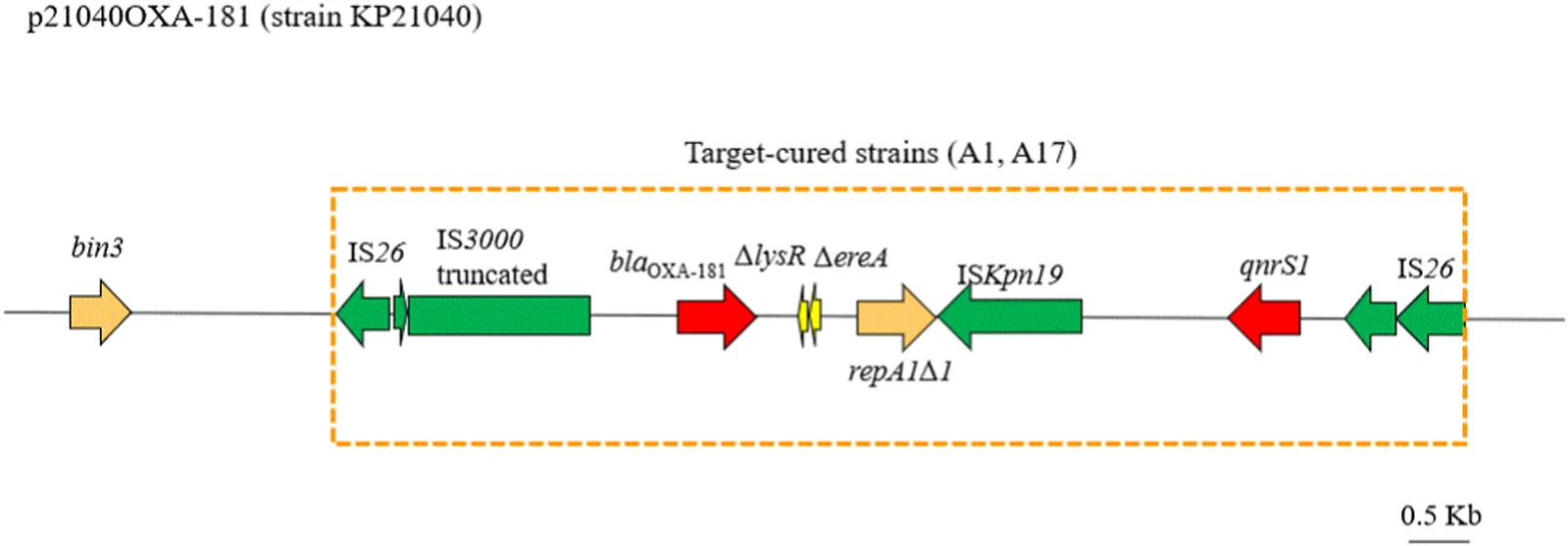
A schematic representation of the deleted region in plasmid p21040OXA-181 from strain KP21040, identified by PCR mapping and sequencing analysis. A deleted segment of 14,124 bp was identified by PCR mapping, indicated by the orange dashed line. This region included two resistance genes—*bla*_OXA-181_ and *qnrS1*—as well as mobile genetic elements insertion sequence (IS) *26*, truncated IS*3000*, and IS*Kpn19*. CRISPR/Cas9-induced double-strand breaks likely triggered IS*26*-mediated recombination, resulting in the excision of the intervening region. This recombination event allowed the flanked segment to evade CRISPR cleavage, thereby preventing complete plasmid elimination and instead leading to the loss of the central resistance gene cluster.

Whole plasmid sequencing of strain KP4-78 revealed a 57,205 bp plasmid, designated p478NDM-1, classified as IncN2 [Supplementary Fig. 7]. In addition to *bla*_NDM-1_, p478NDM-1 carried *int1*, facilitating the integration of resistance gene cassettes into class 1 integrons, as well as other resistance determinants including *aac(6′)-Ib-cr*, *ARR-3*, *aadA16*, *sul1*, and *bla*_OXA-10_. The plasmid also contained multiple insertion sequences, including IS*26*, IS*6100*, IS*91*, and IS*1595*. Following CRISPR/Cas9 treatment, the target-cured strains B1 and B2 showed complete loss of p478NDM-1, as indicated by the disappearance of a ∼55 kb plasmid band in S1-PFGE profiles [Fig. 1]. PCR confirmed the absence of *bla*_NDM-1_, consistent with the removal of the entire plasmid. These findings indicate that CRISPR/Cas9 targeting successfully eliminated the *bla*_NDM-1_–harboring plasmid from KP4-78.

Plasmid sequencing of strain KP5-4 identified three plasmids [Supplementary Fig. 8)] harboring *bla*_KPC-2_: p541KPC-2 (106,581 bp, repB type R1701), p542KPC-2 (198,184 bp, IncFIB), and p543KPC-2 (76,377 bp, IncF). Both p541KPC-2 and p542KPC-2 carried *bla*_KPC-2_, *bla*_TEM-1_, and *tet* (A), with a shared genetic structure (Tn*As1*-*hin*-*klcA*-*bla*_KPC-2_-IS*Kpn27*-*tnpR*) forming a composite transposon-like element adjacent to *bla*_KPC-2_. The third plasmid, p543KPC-2, also carried *bla*_KPC-2_ along with *qnrS1* and *bla*_LAP-2_. After CRISPR/Cas9 intervention, S1-PFGE analysis of target-cured strains C1 and C2 showed complete loss of the three large plasmids (∼80 kb, ∼110 kb, and ∼200 kb). PCR analysis confirmed the absence of *bla*_KPC-2_. The size and gene content of the eliminated plasmids corresponded to those identified in the parental strain, indicating that all three *bla*_KPC-2_–harboring plasmids were successfully removed by CRISPR/Cas9.

## 4 Discussion

Carbapenemase genes are located on plasmids, enabling horizontal transfer to different species and strains. Addressing the spread of plasmid-mediated resistance genes has become an important challenge [20]. A previous study demonstrated that pCasKP-pSGKP could successfully delete the chromosomal resistance gene *bla*_SHV_ [11]. As expected, our results showed the successful removal of these carbapenemase genes, altering the MICs of ertapenem from >32 μg/mL to 0.5 μg/mL (KP4-78 and KP5-4). It suggests that resistance gene curing by pCasKP-pSGKP may be a novel method to resensitize antibiotic susceptibility in clinical CRKP stains.

As predicted, plasmids p478NDM-1, p541KPC-2, p542KPC-2, and p543KPC-2 containing resistance genes were removed by CRISPR. Nonetheless, following modification of *bla*_*OXA-181*_, the complete p2140OXA-181 plasmid remained intact. Instead, a 14,124 bp fragment of the plasmid was deleted. Subsequent examination of the excised gene architecture indicated that *bla*_OXA-181_ was flanked by IS*26* on both sides, truncated IS*3000*, IS*Kpn19*, and the quinolone resistance gene *qnrS1*. IS*26* is prevalent in plasmids and transposons with multiple antibiotic resistance genes, and it facilitates horizontal gene transfer among bacteria, spreading resistance genes to novel hosts [21]. IS*26* insertion in genomes creates clusters of resistance genes, enhancing bacterial antibiotic resistance. Its proximity to resistance gene clusters increases genomic rearrangements, promoting intra-genomic gene distribution. This recombination accelerates the rapid transmission of resistance genes among bacteria, increasing their ability to adapt to antibiotic pressure [22]. IS*26* activity may increase under sustained antibiotic exposure, thereby increasing the likelihood of resistance gene retention in essential genomic sites. This reinforces bacterial resistance to multiple antibiotics. In this study, we showed that IS recombination interfered with pCasKP-pSGKP, hindering plasmid eradication. Sheng et al. reported that natural IS transpositions into cas genes compromised CRISPR-Cas defenses, and that IS transpositions persisted due to DNA repair mechanisms. Moreover, their results emphasized the ability of ISs to undermine CRISPR activity, thereby augmenting bacterial vulnerability to foreign DNA. In a gene editing context, this ability must be carefully regulated to avoid gene editing failure due to IS transpositions into *cas* genes and improve editing outcomes [13]. The identification of anti-CRISPR mechanisms necessitates consideration of both the off-target effects and disruptive influences of host-encoded IS elements on CRISPR/Cas system performance in gene editing, thereby improving editing efficacy and broadening the application of CRISPR/Cas. A strategic approach would involve targeting critical genes or using a CRISPR array with multiple sgRNA cutting sites [23,24]. A previous study demonstrated that an IS-based CRISPR/Cas9 system, termed "pIS26-CRISPR/Cas9," could effectively eliminate plasmid replication and resistance genes. Moreover, the study showed that maintaining the pIS26-CRISPR/Cas9 system in host cells may function as an immune mechanism against the acquisition of exogenous resistance plasmids [25].

To verify the presence and genomic location of resistance determinants prior to CRISPR/Cas9 targeting, comprehensive whole-genome sequencing was performed on the parental strains KP21040, KP4-78, and KP5-4. These analyses, conducted before CRISPR intervention, confirmed that *bla*OXA-181, *bla*NDM-1, and *bla*KPC-2 were intact and plasmid-borne at the time of targeting. Following CRISPR/Cas9 treatment, alterations in plasmid architecture were assessed using S1 nuclease–pulsed-field gel electrophoresis (S1-PFGE) and PCR-based analyses. In KP21040-derived strains A1 and A17, S1-PFGE demonstrated a reproducible reduction in plasmid size relative to the parental strain, consistent with partial plasmid deletion. PCR confirmed the loss of *bla*OXA-181, and subsequent PCR mapping using primers designed from the p21040OXA-181 reference plasmid enabled precise delineation of the deleted region. Sanger sequencing of the resulting amplicons identified an exact IS26–IS26 recombination junction, providing direct molecular evidence for the proposed recombination-mediated deletion mechanism. Notably, the discrete size shift observed by S1-PFGE (approximately 50 kb to 35 kb) indicates that plasmid architecture outside the targeted region remained stable, arguing against large-scale rearrangements or plasmid–chromosome integration events, even in the absence of long-read sequencing for the derivative strains. Similarly, in KP4-78-derived strains B1 and B2, PCR analysis demonstrated the absence of *bla*NDM-1 following CRISPR targeting. Sequencing of the parental KP4-78 strain identified the *bla*NDM-1–harboring plasmid p478NDM-1, and comparative S1-PFGE revealed the loss of a single plasmid band in B1 and B2 corresponding in size to this plasmid, consistent with complete plasmid elimination. Although whole-genome or long-read sequencing was not employed to screen for genome-wide off-target events in CRISPR-edited derivatives, the concordant results obtained from PCR, PFGE, and junction sequencing provide compelling evidence for specific plasmid editing. The high reproducibility of identical deletion or curing profiles across independent derivatives (e.g., A1 and A17) further supports the specificity of CRISPR-Cas9 targeting and argues against major unintended chromosomal rearrangements. Although a formal complementation experiment was not performed, the precise and reproducible concordance between CRISPR-mediated loss of resistance gene–containing regions (confirmed by PCR mapping and S1-PFGE) and the immediate, substantial reduction in MICs supports a direct functional link between plasmid or gene deletion and restored antimicrobial susceptibility. Collectively, these results demonstrate that CRISPR/Cas9 effectively eliminated targeted resistance genes and their associated plasmids, as validated by an integrated combination of genome sequencing, PFGE, and PCR-based mapping approaches.

In our study, CRISPR/Cas9-mediated targeting of the *bla*_OXA-181_ gene unexpectedly resulted in the deletion of an approximately 15 kb region within the p2140OXA-181 plasmid. Rather than being the result of off-target activity due to suboptimal sgRNA design, we propose that this deletion was facilitated by the presence of ISs, particularly IS*26*, flanking the *bla*_OXA-181_ locus. IS*26* elements are well known to mediate homologous recombination, especially in the presence of double-strand DNA breaks. We hypothesize that the Cas9-induced cleavage initiated a recombination event between these IS*26* sequences, resulting in the excision of the intervening segment harboring both *bla*_OXA-181_ and *qnrS1*. In addition, complete loss of resistance plasmids such as p478NDM-1, p541KPC-2, p542KPC-2, and p543KPC-2 in multiple CRKP isolates can be attributed to Cas9-mediated double-strand breaks. This observation is consistent with previous reports demonstrating that targeted cleavage of essential plasmid regions by Cas9 can destabilize and eliminate plasmids in Gram-negative bacteria [26]. It is important to distinguish between CRISPR/Cas9-induced plasmid elimination and the subsequent plasmid curing procedures employed in our workflow. Plasmid curing was performed only after CRISPR/Cas9 successfully disrupted the target plasmids and served solely to remove the CRISPR delivery vectors, which encode Cas9 and sgRNA. Therefore, the observed elimination of resistance plasmids in this study is a direct consequence of CRISPR/Cas9 activity rather than spontaneous or non-specific plasmid loss. Our results further demonstrate the high specificity of the CRISPR/Cas9 system in the KP5-4 strain, where p541KPC-2, p542KPC-2, and p543KPC-2 were successfully eliminated. This underscores the system's capacity to precisely target and disrupt antimicrobial resistance genes in complex multidrug-resistant bacterial backgrounds. Such precision is crucial for potential clinical applications, where targeted plasmid removal can restore the efficacy of key antibiotics such as carbapenems and help curb the spread of resistance genes among pathogenic bacterial populations [25,27]. Furthermore, the ability of this system to eliminate multiple resistance plasmids within a single strain highlights its potential as a powerful tool against multidrug-resistant organisms. Interestingly, sequencing of p543KPC-2 revealed the presence of the *qnrS1* gene. Following successful removal of this plasmid in the target-cured strains (C1, C2), restored susceptibility to levofloxacin was anticipated. However, both derivatives remained resistant, comparable to the parental KP5-4 isolate. Although chromosomal sequencing (e.g., of *gyrA* or *parC*) was not performed in this study, as our primary focus was on plasmid-borne resistance, the persistence of levofloxacin resistance despite the complete elimination of the *qnrS1*-carrying plasmid suggests that resistance in this clinical isolate is predominantly mediated by chromosomal mechanisms, such as mutations within quinolone resistance–determining regions or efflux pump overexpression.

We demonstrated that targeting a gene segment flanked by IS*26* enabled recombination to bypass CRISPR-induced double-strand breaks, resulting in the deletion of a ∼15 kb fragment carrying both *bla*_OXA-181_ and *qnrS1*. This simultaneously disrupted carbapenem and quinolone resistance. Notably, the same CRISPR system also achieved complete elimination of all resistance plasmids in *bla*_KPC-2_ harboring CRKP strains. These findings highlight the dual functionality of the pCasKP-pSGKP platform in mediating both targeted gene disruption and whole-plasmid curing, even in the presence of mobile genetic elements such as IS*26*. While the plasmid backbone itself is not novel, our study reveals a previously unrecognized mechanism of IS*26* facilitated CRISPR-mediated deletion and underscores the therapeutic promise of this approach for multidrug-resistant CRKP. A key limitation for clinical translation remains the lack of efficient *in vivo* delivery methods for CRISPR-Cas9. Another limitation of this study is the absence of systematic assessments of bacterial fitness, competitive dynamics, and long-term stability following plasmid curing. Although CRISPR-cured strains showed stable *in vitro* growth and reproducible restoration of antibiotic susceptibility during the observation period, potential fitness costs, compensatory mutations, and long-term persistence of the cured state cannot be excluded and warrant further investigation.

## 5 Summary

Our results show that the CRISPR-Cas9–mediated pCasKP-pSGKP system can eliminate plasmid-encoded carbapenemase genes in clinical CRKP isolates and, in specific genetic contexts, facilitate the concurrent removal of associated quinolone resistance determinants. Notably, the presence of flanking insertion sequences enabled recombination-driven deletion following CRISPR-induced double-strand breaks, thereby shaping the structural outcome of plasmid editing. These findings support CRISPR-Cas9–based gene and plasmid curing as a proof-of-concept strategy for addressing plasmid-mediated carbapenem resistance in Gram-negative pathogens.

## Authors’ contributions

SFK and CHL designed and coordinated this study and wrote the manuscript. TYH, FJC, and CYL contributed to carrying out the experiments. All authors read and approved the final manuscript.

## Availability of data and materials

The data are available for any scientific use from the corresponding author upon reasonable request.

## Consent for publication

Not applicable.

## Funding

This work was supported by the Research Foundation of the 10.13039/501100012283Chiayi Chang Gung Memorial Hospital (CMRPG6P0011-12 and CMRPG6P0081).

## References

[1] Wyres KL, Holt KE. (2018). *Klebsiella pneumoniae* as a key trafficker of drug resistance genes from environmental to clinically important bacteria. Curr Opin Microbiol.

[2] Guo MQ, Wang YT, Wang SS, Chen LK, Xu YH, Li G. (2024). Genomic epidemiology of hypervirulent carbapenem-resistant *Klebsiella pneumoniae* at jinshan local hospital, Shanghai, during 2014-2018. J Microbiol Immunol Infect.

[3] Halat DH, Moubareck CA. (2020). The current burden of carbapenemases: review of significant properties and dissemination among gram-negative bacteria. Antibiotics (Basel).

[4] Buckner MMC., Ciusa ML, Piddock LJV. (2018). Strategies to combat antimicrobial resistance: anti-plasmid and plasmid curing. FEMS Microbiol Rev.

[5] Mojica FJM, Díez-Villaseñor C, García-Martínez J, Soria E. (2005). Intervening sequences of regularly spaced prokaryotic repeats derive from foreign genetic elements. J Mol Evol.

[6] Hsu PD, Lander ES, Zhang F. (2014). Development and applications of CRISPR-Cas9 for genome engineering. Cell.

[7] Doudna JA, Charpentier E. (2014). Genome editing. The new frontier of genome engineering with CRISPR-Cas9. Science.

[8] Gholizadeh P, Köse Ş, Dao S, Ganbarov K, Tanomand A, Dal T (2020). How CRISPR-Cas system could be used to combat antimicrobial resistance. Infect Drug Resist.

[9] Bikard D, Hatoum-Aslan A, Mucida D, Marraffini LA. (2012). CRISPR interference can prevent natural transformation and virulence acquisition during in vivo bacterial infection. Cell Host Microbe.

[10] Sun Q, Wang Y, Dong N, Shen L, Zhou H, Hu Y (2019). Application of CRISPR/Cas9-based genome editing in studying the mechanism of pandrug resistance in *Klebsiella pneumoniae*. Antimicrob Agents Chemother.

[11] Wang Y, Wang S, Chen W, Song L, Zhang Y, Shen Z (2018). CRISPR-Cas9 and CRISPR-assisted cytidine deaminase enable precise and efficient genome editing in *Klebsiella pneumoniae*. Appl Environ Microbiol.

[12] Wang Q, Liu Y, Chen R, Zhang M, Si Z, Wang Y (2024). Genomic insights into the evolution and mechanisms of carbapenem-resistant hypervirulent *Klebsiella pneumoniae* co-harboring *bla*_KPC_ and *bla*_NDM_: implications for public health threat mitigation. Ann Clin Microbiol Antimicrob.

[13] Sheng Y, Wang H, Ou Y, Wu Y, Ding W, Tao M (2023). Insertion sequence transposition inactivates CRISPR-Cas immunity. Nat Commun.

[14] Mlynarcik P, Roderova M, Kolar M. (2016). Primer evaluation for PCR and its application for detection of carbapenemases in Enterobacteriaceae. Jundishapur J Microbiol.

[15] Wayne PCLSI. (2024).

[16] Diancourt L, Passet V, Verhoef J, Grimont PAD, Brisse S. (2005). Multilocus sequence typing of *Klebsiella pneumoniae* nosocomial isolates. J Clin Microbiol.

[17] Brisse S, Fevre C, Passet V, Issenhuth-Jeanjean S, Tournebize R, Diancourt L (2009). Virulent clones of *Klebsiella pneumoniae*: identification and evolutionary scenario based on genomic and phenotypic characterization. PLoS One.

[18] Hao M, He Y, Zhang H, Liao XP, Liu YH, Sun J (2020). CRISPR-Cas9-mediated carbapenemase gene and plasmid curing in carbapenem-resistant Enterobacteriaceae. Antimicrob Agents Chemother.

[19] Chen C, Yang F, Chen M, Xu Y, Xie Y, Liu R (2024). ST11 KPC-2-Producing *Klebsiella pneumoniae* isolated from patient with acute myelocytic leukemia. Infect Drug Resist.

[20] Hardiman CA, Weingarten RA, Conlan S, Khil P, Dekker JP, Mathers AJ (2016). Horizontal transfer of carbapenemase-encoding plasmids and comparison with hospital epidemiology data. Antimicrob Agents Chemother.

[21] Sun D, Sun X, Hu Y, Yamaichi Y. (2023). Editorial: horizontal gene transfer mediated bacterial antibiotic resistance, volume II. Front Microbiol.

[22] Moser KA, Zhang L, Spicknall I, Braykov NP, Levy K, Marrs CF (2018). The role of mobile genetic elements in the spread of antimicrobial-resistant *Escherichia coli* from chickens to humans in small-scale production poultry operations in rural Ecuador. Am J Epidemiol.

[23] Wang P, He D, Li B, Guo Y, Wang W, Luo X (2019). Eliminating mcr-1-harbouring plasmids in clinical isolates using the CRISPR/Cas9 system. J Antimicrob Chemother.

[24] Lauritsen I, Porse A, Sommer MOA, Nørholm MHH. (2017). A versatile one-step CRISPR-Cas9 based approach to plasmid-curing. Microb Cell Fact.

[25] He YZ, Kuang X, Long TF, Li G, Ren H, He B (2022). Re-engineering a mobile-CRISPR/Cas9 system for antimicrobial resistance gene curing and immunization in *Escherichia coli*. J Antimicrob Chemother.

[26] Jiang Y, Chen B, Duan C, Sun B, Yang J, Yang S. (2015). Multigene editing in the *Escherichia coli* genome via the CRISPR-Cas9 system. Appl Environ Microbiol.

[27] Tao S, Chen H, Li N, Fang Y, Zhang H, Xu Y (2023). Elimination of blaKPC-2-mediated carbapenem resistance in *Escherichia coli* by CRISPR-Cas9 system. BMC Microbiol.

