# Supplementary material for: CRISPR-Cas9-mediated elimination of plasmid-borne carbapenemase genes restores ertapenem susceptibility in clinical Klebsiella pneumoniae isolates
Source: Biomed J. 2026 Mar 16;49(6):100966. doi: 10.1016/j.bj.2026.100966 (PMC13769451; doi:10.1016/j.bj.2026.100966)
Supplement: Supplementary file 1 [file mmc1.docx]

**Supplementary Table 1.** Primers used for MLST of *K. pneumoniae.*

| **Target gene** | **Primers name** | **sequences** | **Reference** |
| --- | --- | --- | --- |
| *rpoB* | Vic3 | GGCGAAATGGCWGAGAACCA | [16] [17] |
|  | Vic2 | GAGTCTTCGAAGTTGTAACC |  |
| *rpoB* | Vic3-F | GGCGAAATGGCWGAGAACCA | [16] [17] |
|  | Vic2-R | GAGTCTTCGAAGTTGTAACC |  |
| *gapA* | gapA173-F | TGAAATATGACTCCACTCACGG | [16] [17] |
|  | gapA181-R | CTTCAGAAGCGGCTTTGATGGCTT |  |
| *mdh* | mdh130-F | CCCAACTCGCTTCAGGTTCAG | [16] [17] |
|  | mdh867-R | CCGTTTTTCCCCAGCAGCAG |  |
| *pgi* | pgi1F | GAGAAAAACCTGCCTGTACTGCTGGC | [16] [17] |
|  | Pgi1R | CGCGCCACGCTTTATAGCGGTTAAT |  |
| *phoE* | phoE604.1-F | ACCTACCGCAACACCGACTTCTTCGG | [16] [17] |
|  | phoE604.1-R | TGATCAGAACTGGTAGGTGAT |  |
| *infB* | infB1F | CTCGCTGCTGGACTATATTCG | [16] [17] |
|  | infB1R | CGCTTTCAGCTCAAGAACTTC |  |
| *tonB* | tonB1F | CTTTATACCTCGGTACATCAGGTT | [16] [17] |
|  | tonB1R | ATTCGCCGGCTGRGCRGAGAG |  |

**Supplementary Table 2.** List of CRISPR plasmids

| **Plasmid** | **Description** | **GenBank number** |
| --- | --- | --- |
| pCasKP-apr | Thermosensitive plasmid, expresses Cas9 and lambda red proteins in *K. pneumoniae* (Apr^r^) | MH587687.1 |
| pCasKP-hph | Thermosensitive plasmid, expresses Cas9 and lambda red proteins in *K. pneumoniae* (Hyg^r^) | MH587688.1 |
| pSGKP-km | Plasmid expressing sgRNA in *K. pneumoniae* (Km^r^) | MH587683.1 |
| pSGKP-spe | Plasmid expressing sgRNA in *K. pneumoniae* (spe^r^) | MH587684.1 |

**Supplementary Table 3.** Reagents, materials, and equipment used in this study.

| Item | Catalog No. /GenBank | Manufacturer / Source |
| --- | --- | --- |
| QIAamp DNA Mini Kit | 69540 | QIAGEN, Germany |
| LB Broth | 12780-052 | Invitrogen™, USA |
| Tryptone Soy Broth | 211768 | BD Diagnostic Systems, MD, USA |
| Proteinase K | – | Sigma-Aldrich, USA |
| Apramycin | – | Sigma-Aldrich, USA |
| Kanamycin | – | Sigma-Aldrich, USA |
| BsaI Restriction Enzyme | – | New England Biolabs (NEB), USA |
| T4 DNA Ligase | – | New England Biolabs (NEB), USA |
| QIAGEN Plasmid Maxi Kit | 12162 | QIAGEN, Germany |
| S1 Nuclease | – | Thermo Scientific™, Lithuania |
| Electroporation Apparatus | Gene Pulser Xcell™ | Bio-Rad Laboratories, USA |
| E-test Ertapenem | – | bioMérieux, France |
| E-test Levofloxacin | – | bioMérieux, France |
| MALDI-TOF MS Biotyper | Microflex LT/SH | Bruker Daltonics GmbH, Germany |
| BD Phoenix™ AST System | M50 | BD Diagnostic Systems, Sparks, MD, USA |
| E. coli DH5α | – | YB Biotech, Taiwan |
| pCasKP-apr Plasmid | Addgene #117231; GenBank MH587687.1 | [https://www.addgene](https://www.addgene/) |
| pCasKP-hph Plasmid | Addgene #117232; GenBank MH587688.1 | [https://www.addgene](https://www.addgene/) |
| pSGKP-km Plasmid | Addgene #117233; GenBank MH587683.1 | [https://www.addgene](https://www.addgene/) |
| pSGKP-spe Plasmid | Addgene #117234; GenBank MH587684.1 | [https://www.addgene](https://www.addgene/) |
