# Supplementary material for: CRISPR-Cas9-mediated elimination of plasmid-borne carbapenemase genes restores ertapenem susceptibility in clinical Klebsiella pneumoniae isolates
Source: Biomed J. 2026 Mar 16;49(6):100966. doi: 10.1016/j.bj.2026.100966 (PMC13769451; doi:10.1016/j.bj.2026.100966)
Supplement: Supplementary file 2 [file mmc2.docx]

**Supplementary Fig. 1**. Schematic of CRISPR/Cas9 targeting and spacer–PAM relationship. This conceptual diagram illustrates the interaction between the CRISPR/Cas9 complex and double-stranded DNA. The 20-nucleotide spacer in the sgRNA binds to its complementary sequence adjacent to a protospacer adjacent motif (PAM; 5'-NGG-3'). Cas9 introduces double-strand break three nucleotides upstream of the PAM site. Arrows indicate transcriptional direction and strand polarity.

**Supplementary Fig. 2**. Sequence-specific targeting of *bla*_OXA-181_ by sgRNA OXA181-F1. The 20-nt spacer of sgRNA OXA181-F1 specifically binds the *bla*_OXA-181_ gene sequence. The adjacent protospacer adjacent motif (PAM) sequence (5’-NGG-3’) is essentials for Cas9 recognition. Cas9 cleaves the DNA at the target site, enabling plasmid disruption. Base pairing and directionality are indicated.

**Supplementary Fig. 3.** Sequence-specific targeting of the *bla*_KPC-2_ by sgRNA KPC-F1. The sgRNA KPC-F1 contains a 20-nt spacer targeting the bla_KPC2_ gene, positioned adjacent to a canonical protospacer adjacent motif (PAM; 5'-NGG-3'). This enables Cas9-mediated cleavage. The figure shows complementary pairing between the sgRNA and target DNA, as well as the cleavage site.

**Supplementary Fig. 4.** Targeting of bla_NDM-1_ by sgRNA and cleavage site illustration. This schematic depicts the base pairing between sgRNA and the **bla_NDM-1_** gene. The protospacer adjacent motif (PAM; 5'-NGG-3') site is highlighted in red, and the predicted Cas9 cleavage occurs three nucleotides upstream. Spacer-target alignment and strand orientation are shown.

**Supplementary Fig. 5.** Antimicrobial susceptibility of wild-type and target-cured CRKP strains. Strain KP21040 showed resistance to ertapenem (MIC: 2 μg/mL) and levofloxacin (MIC: 1.5 μg/mL). The target-cured strain A1 exhibited enlarged inhibition zones and significantly reduced MICs for both drugs, indicating restored susceptibility. KP4-78 and KP5-4 had ertapenem MICs ≥ 32 μg/mL, while target-cured strains B1 and C1 showed dramatically reduced MICs (0.5 μg/mL), indicating successful CRISPR-mediated resensitization.

**Supplementary Fig. 6.** Partial deletion of p21040OXA-181 following CRISPR targeting. The 51,479 bp IncX3-type plasmid p21040OXA-181 was not entirely removed following CRISPR intervention targeting bla_OXA-181_. Instead, a gene segment flanked by IS*26*, including the central region containing bla_OXA-181_ and qnrS1, was excised via IS*26*-mediated recombination.

**Supplementary Fig. 7.** Genetic features of plasmid p478NDM-1. p478NDM-1 is a 57,205 bp IncN2 plasmid carrying a class 1 integron (IntI1), which mediates integration of resistance cassettes including aac(6')-Ib-cr, ARR-3, aadA16, and sul1. It encodes two carbapenemase genes (bla_NDM-1_ and bla_OXA-10_).

**Supplementary Fig. 8.** Genomic structure of KPC-2–carrying plasmids. p541KPC-2 (106,581 bp, repB-type), p542KPC-2 (198,184 bp, IncFIB-type), and p543KPC-2 (76,377 bp, IncF-type) all carry bla_KPC-2_. p541KPC-2 and p542KPC-2 also contain bla_TEM-1_and tet(A), with shared transposon architecture (TnAs1-hin-klcA-bla_KPC-2_-IS*Kpn27*-*tnpR*). p543KPC-2 carries additional resistance genes qnrS1 and bla_LAP-2_.
